# Современные представления о генетических и иммуногистохимических особенностях пролактин-секретирующих аденом гипофиза

**DOI:** 10.14341/probl13222

**Published:** 2023-06-30

**Authors:** А. С. Шутова, Л. К. Дзеранова, С. Ю. Воротникова, М. A. Кутин, Е. А. Пигарова

**Affiliations:** Национальный медицинский исследовательский центр эндокринологии; Национальный медицинский исследовательский центр эндокринологии; Национальный медицинский исследовательский центр эндокринологии; Национальный медицинский исследовательский центр нейрохирургии имени академика Н.Н. Бурденко; Национальный медицинский исследовательский центр эндокринологии

**Keywords:** аденомы гипофиза, пролактиномы, резистентность к терапии, иммуногистохимическое исследование, генетическое исследование

## Abstract

Пролактиномы — наиболее распространенные гормонально-активные аденомы гипофиза. В 20% случаев наблюдается резистентность к методу выбора их лечения — медикаментозной терапии агонистами дофамина. Наличие резистентности обусловливает прогрессию патологических проявлений гиперпролактинемии и негативные топографо-анатомические изменения пролактиномы. Причины нечувствительности к терапии агонистами дофамина не вполне изучены, а подходы к ведению пациентов требуют уточнения. Существующие в настоящее время концепции резистентности базируются на данных, получаемых в результате оперативного вмешательства или после периода длительной неэффективной терапии. В связи с этим весьма актуален поиск методов оценки чувствительности пролактин-секретирующих аденом к медикаментозной терапии на раннем диагностическом этапе. Особое место среди этих методов занимают молекулярно-генетические исследования, позволяющие прогнозировать ответ аденомы на медикаментозную терапию до проведения хирургического лечения. На основании полученных данных возможно формирование персонализированного алгоритма ведения пациентов.

## Abstract (en)

Prolactinomas are the most common secreting adenomas of the pituitary. In 20% of cases resistance to dopamine-agonists treatment is observed. Medical therapy resistance causes progression of pathological symptoms of hyperprolactinemia and negative topographic and anatomical changes of prolactinoma. The causes of ineffectiveness of dopamine agonists therapy are not fully understood as well as approaches to managing patients require clarification. Current concepts of resistance are based on the data obtained as a result of surgery or after a period of long-term ineffective therapy. Thus, it is very important to find methods of assessing the sensitivity of prolactin-secreting adenomas to drug therapy before surgical treatment. Genetic and immunohistochemical studies find special place among these methods, making it possible to predict adenoma’s response to drug therapy at early diagnostic stage. Obtained results will allow us to form personalized algorithm for managing patients.

## ВВЕДЕНИЕ

Аденомы гипофиза представляют собой гетерогенную группу новообразований, отличающихся по секреторному потенциалу, топографо-анатомическим характеристикам, клиническим проявлениям. В соответствии с функциональной активностью различают гормонально-активные, неактивные и так называемые «молчащие» аденомы, способность к гормональной секреции которых определяется только по данным иммуногистохимического (ИГХ) исследования. Среди всех гормонально-активных аденом гипофиза чаще встречаются пролактин-секретирующие, составляя около 53% всех новообразований гипоталамо-гипофизарной области [1]. Клиническая картина пролактином складывается из двух составляющих: проявлений гормональной гиперпродукции (таких как гипогонадизм, гинекомастия, галакторея и бесплодие) и масс-эффекта опухоли, приводящего к возникновению очаговой неврологической симптоматики, зрительных нарушений — вплоть до слепоты вследствие атрофии зрительного нерва, апоплексии гипофиза. Основной способ лечения пролактином, в отличие от прочих аденом гипофиза, при которых выполняется хирургическое вмешательство, заключается в назначении медикаментозной терапии агонистами дофамина (АД) [2][3]. Терапия АД в большинстве случаев позволяет достичь антисекреторного и антипролиферативного эффекта в виде нормализации уровня пролактина в сыворотке крови и уменьшения размеров опухоли. Однако около 20% пациентов с пролактин-секретирующими аденомами гипофиза неудовлетворительно реагируют даже на высокие дозы АД, что обусловлено резистентностью к терапии [4]. Следствие длительного периода неэффективной медикаментозной терапии — неизбежная прогрессия патологических изменений, вызванных стойкой гиперпролактинемией. Кроме того, отсутствие патогенетического лечения способствует отрицательной динамике размеров аденомы в виде увеличения ее объема и распространения вне области турецкого седла. Данные изменения ухудшают результаты потенциального хирургического лечения и увеличивают риск как интра-, так и послеоперационных осложнений. Угрожающими жизни осложнениями неэффективного консервативного лечения, требующими проведения безотлагательного хирургического вмешательства, могут быть ликворея и кровоизлияние в опухоль [5][6].

Выявление этиопатогенетических маркеров нечувствительности пролактином к терапии, использование которых позволит определить оптимальную тактику ведения данной группы пациентов, крайне актуально.

## Эволюция концепций резистентности пролактином к медикаментозной терапии

Предпринималось множество попыток классифицировать резистентность пролактин-секретирующих аденом к терапии АД. В результате сформировалось понятие резистентности как отсутствия нормализации уровня пролактина сыворотки крови и уменьшения объема аденомы на 50% и более от исходного при приеме максимально переносимых доз АД, но не менее 3 мг каберголина в неделю, на фоне непрерывного полугодового лечения [7][8]. Необходимо отметить, что в ряде случаев наблюдается недостаточная эффективность терапии АД — достижение уменьшения размеров опухоли без нормализации уровня пролактина или обратная ситуация — снижение уровня пролактина без уменьшения размеров аденомы, что обусловливает необходимость учета обоих критериев при определении понятия резистентности пролактином к терапии. Полная резистентность проявляется отсутствием какого-либо значимого эффекта от назначения агонистов дофамина в отношении как гормональной секреции, так и объема опухолевой ткани, частичная наблюдается при снижении секреции пролактина без нормализации его уровня или уменьшения размеров аденомы, но менее чем 50% от исходного [8].

В настоящее время парадигма резистентности к лечению АД включает в себя ряд положений. Резистентные пролактиномы зачастую демонстрируют признаки клинико-морфологической «агрессивности» — обладают большим размером (10 мм и более), плотно гранулированным типом строения, склонностью к инвазивному росту, более высоким уровнем ki-67 [9]. Отмечены характерные изменения рецепторного аппарата пролактин-секретирующих аденом, резистентных к терапии: уменьшение количества дофаминовых D2-рецепторов, снижение синтеза протеина G, обеспечивающего связывание агонистов дофамина с D2-рецептором, изменение количества эстрогеновых рецепторов (ERα), нарушение трансмембранной передачи сигнала D2-рецепторов [10].

Анализ вышеуказанных характеристик возможен только на этапе получения гистологического операционного материала, в связи с чем особую актуальность приобретает поиск методов, которые позволят прогнозировать ответ аденомы на медикаментозное лечение до проведения оперативного вмешательства. Особое место среди таких методов занимают молекулярно-генетические исследования.

## Патогенетические особенности этапов дифференцировки гипофиза в туморогенезе пролактин-секретирующих аденом

Пролактиномы имеют моноклональную природу и могут возникать вследствие генетических нарушений на одном из этапов развития гипофиза — в процессе эмбриогенеза, на стадии дифференцировки клеточных линий или непосредственно в ткани зрелого органа. Понимание молекулярно-генетической природы возникновения пролактином невозможно без детального изучения этапов эмбриогенеза и формирования гипофиза.

Гипофиз состоит из двух долей — адено- и нейрогипофиза, имеющих принципиально разные источники развития и объединенных исключительно топографической близостью. Аденогипофиз — производное эпителия оральной эктодермы (первичного рта), в то время как задняя доля гипофиза развивается из нейроэпителия промежуточного мозга или нейроэктодермы. Зачаток нейрогипофиза, расширяясь в вентральном направлении, приводит к инвагинации эпителия первичного рта и формированию кармана Ратке (рис. 1, а, б). Последующее отсоединение этого участка от оральной эктодермы (рис. 1, в) является отправной точкой формирования аденогипофиза и дифференцировки его клеточных линий (рис. 1, г) [11].

Необходимо отметить, что каждый этап развития гипофиза регулируется определенным транскрипционным фактором или группой факторов. Так, начальный этап эмбриогенеза — сближение нейроэктодермы и эпителия первичного рта находится под регуляторным влиянием факторов транскрипции Nkx2.1, Sox3 и Lhx2 (рис. 1, а). Следующими в процесс формирования гипофиза вступают транскрипционные факторы семейства Pitx — Pitx1 и Pitx2, а также Lhx3-4 (рис. 1, б, в), которые способствуют взаимному сближению предшественников обеих долей гипофиза и возникновению кармана Ратке. Появление факторов Hesx1 и Prop-1 завершает этап эмбриогенетического каскада — разрыв связи кармана Ратке с эпителием первичного рта и приобретение автономности передней долей гипофиза (рис. 1, г) [11][12].

**Figure:**
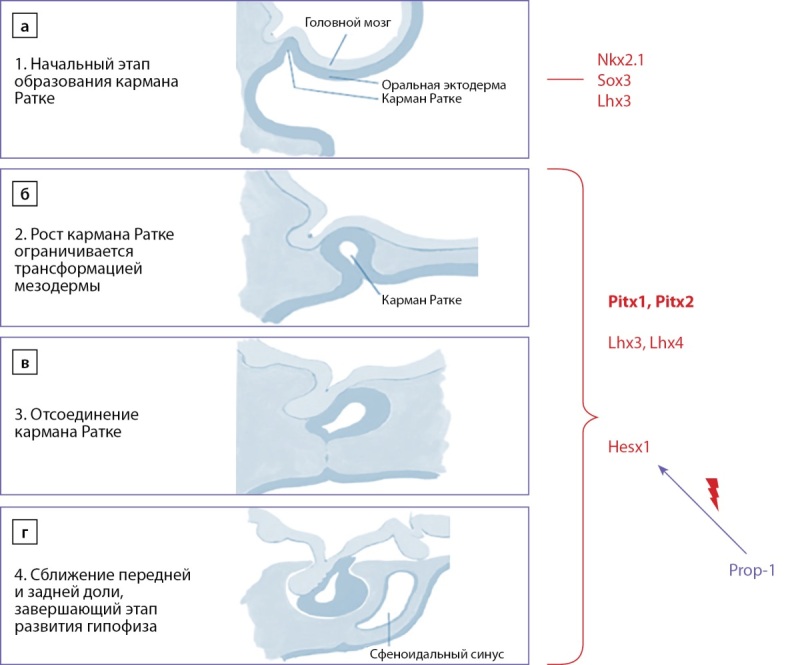
Рисунок 1. Молекулярно-генетическая регуляция эмбриогенеза гипофиза (адаптировано из «The Netter collection of medical illustrations: Endocrine system, Volume 2, Second Edition»).Figure 1. Molecular genetic regulation of pituitary embryogenesis (adapted from The Netter collection of medical illustrations: Endocrine system, Volume 2, Second Edition)

Изменение последовательности взаимодействия транскрипционных факторов, их недостаточная или несвоевременная экспрессия приводят к изменениям последовательности генов. Возникшие мутации индуцируют туморогенез посредством различных механизмов: активации протоонкогенов, подавления генов-супрессоров опухолевого роста, нарушения работы белков-регуляторов клеточного цикла [12]. Впоследствии происходит опухолевая трансформация клетки c приобретением избирательного пролиферативного преимущества и дальнейшей клональной экспансией, приводящей к образованию пролактиномы [12–14].

Дифференцировка клеточных линий аденогипофиза происходит под контролем факторов транскрипции, аффилированных к определенному типу клеток (рис. 2).

**Figure:**
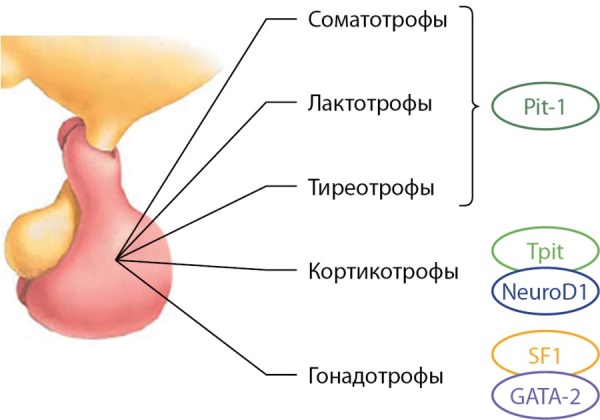
Рисунок 2. Регуляция дифференцировки клеточных линий аденогипофиза под воздействием транскрипционных факторов.Pit-1 — гипофиз-специфичный транскрипционный фактор 1 (pituitary-specific transcription factor 1), Tpit — T-box транскрипционный фактор TBX19 (T-box transcription factor TBX19), GATA-2 — GATA связывающий белок 2 (GATA binding protein 2), SF-1 — стероид-специфичный транскрипционный фактор 1 (steroidogenic factor 1).Figure 2. Regulation of differentiation of adenohypophysis cell lines under the influence of transcription factors

Наиболее важным фактором транскрипции, в отсутствие которого невозможна дифференцировка лактотрофов, соматотрофов и тиреотрофов, является Pit-1. Нарушения экспрессии этого транскрипционного фактора, возникшие в период дифференцировки клеточных линий гипофиза, могут приводить к формированию пролактин-секретирующей аденомы с моно- или плюригормональным типом секреции (табл. 1).

**Table:** Табл. 1. Соответствие типа гормональной активности аденом гипофиза и характерных факторов транскрипцииTab. 1. Correspondence between the type of hormonal activity of pituitary adenomas and characteristic transcription factors Примечание. Pit-1 — гипофиз-специфичный транскрипционный фактор 1 (pituitary-specific transcription factor 1), ER-α — эстрогеновый рецептор альфа, GATA-2 — транскрипционный фактор, участвующий в регуляции процессов эмбриогенеза. ПРЛ — пролактин; СТГ — соматотропный гормон; ТТГ — тиреотропный гормон.

Опухоль	Транскрипционные факторы	Гормональная активность
Pit-1-позитивные опухоли
Лактотрофные аденомы
Редко гранулированные	Pit-1, ER-α	ПРЛ, α-субъединица
Плотно гранулированные	ПРЛ
Ацидифильные аденомы из стволовых клеток	ПРЛ, СТГ
Соматотрофные аденомы
Редко гранулированные	Pit-1	СТГ, слабая экспрессия
Плотно гранулированные	СТГ, α-субъединица
Маммосоматотрофные аденомы	Pit-1, ER-α	СТГ, ПРЛ, α-субъединица
Смешанные СТГ-ПРЛ-секретирующие аденомы
Плюригормональные аденомы, секретирующие СТГ	Pit-1, ER-α	СТГ, ПРЛ, α-субъединица, β-ТТГ
Тиреотрофные аденомы
Тиреотрофные аденомы	Pit-1, GATA-2	β-ТТГ, α-субъединица

Изменения, приводящие к возникновению клона аденоматозных лактотрофных клеток, могут произойти и в клетках зрелого гипофиза. Пролактиномы, как и другие аденомы гипофиза, сохраняют способность подчиняться действию транскрипционных факторов и ко-факторов. Так, пролактин-секретирующие аденомы регулируются Pit-1 и ER-α, соматотропиномы — Pit-1 и GhRh-R, тиреотропиномы — Pit-1 и GATA-2. Этиологическая общность пролактин-, СТГ- и ТТГ-секретирующих (СТГ — соматотропный гормон, ТТГ — тиреотропный гормон) аденом гипофиза и наличие единого транскрипционного фактора — Pit-1 являются причинами образования аденом с сочетанной секрецией (табл. 1). Важно подчеркнуть, что клетки аденогипофиза даже в зрелом состоянии проявляют значительную пластичность, изменяя гормональную активность в соответствии с функциональными потребностями организма. В частности, обратимая трансдифференцировка существует между членами Pit-1 группы: соматотрофы трансформируются в лактотрофы во время беременности или в тиреотрофы в случае гипотиреоза [12][15].

Тесное взаимодействие клеток аденогипофиза не ограничивается адаптивной дифференцировкой. Показано, что клетки, относящиеся к единой линии развития, формируют клеточные сети трехмерного строения. Установление плотного межклеточного контакта происходит вследствие движения выростов цитоплазмы — цитонем — в направлении соседней клетки. Трехмерная организация гомотипичных клеток аденогипофиза обеспечивает стремительное изменение секреторнoй активности в зависимости от актуальных потребностей организма. Координированный ответ всех клеток сети достигается, в том числе, путем изменения интенсивности локального кровотока [15]. Этот механизм имеет важное значение в контексте рассмотрения патогенеза пролактином: нарушение согласованности передачи сигнала и неадекватное перераспределение кровоснабжения внутри трехмерной системы лактотрофов способствуют приобретению отдельным клеточным пулом пролиферативного преимущества с последующим формированием аденоматозного клона.

## Сохранение потенциала дифференцировки стволовых клеток в ткани зрелого гипофиза

Интересный результат продемонстрирован в исследовании, посвященном изучению плюригормональных клеток аденогипофиза взрослых людей [16]. В работе выявлена сохраняющаяся ко-экспрессия пролактина, CТГ и ТТГ данным типом клеток. Полученные данные свидетельствуют, что в аденогипофизе в течение всей жизни сохраняется определенный пул плюригормональных клеток, способных стать источником развития аденом при воздействии пусковых факторов [16]. Подтверждение этому обнаруживается в исследованиях Т. Fauquier, К. Rizzoti, С. Andoniadou. Авторы подтвердили наличие в ткани зрелого гипофиза стволовых клеток — прогениторов, обладающих потенциалом дифференцировки в большинство гормонально-активных клеточных линий. Важно отметить, что в этих клетках выявлена экспрессия ключевого фактора транскрипции стволовых клеток — эмбриогенетического маркера гипофиза Sox2 [17].

В зрелом органе этот транскрипционный фактор обнаруживается в области расщелины гипофиза, что соотносится с сохранением пула стволовых клеток. Увеличение экспрессии Sox2 в зрелом гипофизе может свидетельствовать о повышении его неопластического потенциала [18].

Необходимо подчеркнуть, что поиск характерных молекулярно-генетических особенностей, установление влияния и определение роли стволовых и клеток-прогениторов в формировании пролактином имеют ключевое значение для прогнозирования пути развития аденомы и ее ответа на различные виды лечения.

## Генетические аспекты пролактином, резистентных к терапии агонистами дофамина

Генетические нарушения, свойственные гормонально-активным аденомам гипофиза, в том числе пролактиномам, в большей степени остаются неизвестными. Небольшая часть аденом имеет четкую генетическую причину и входит в состав одного из синдромов: МЭН-1 — синдрома множественных эндокринных неоплазий 1-го типа (мутация или делеция в гене MEN1, находящегося на хромосоме в локусе 11q13), комплекса Карни (мутация в I-a-регуляторной субъединице гена супрессора протеинкиназы типа А, локализующегоcя на хромосомах 17q24 и 2р16 (PRKAR1A ген), семейных изолированных аденом гипофиза — FIPA (мутация в гене AIP). Также описаны редкие случаи МЭН-1-подобного синдрома, ассоциированного с пролактиномами, развивающегося вследствие мутации в гене циклинзависимой киназы р21 (ген CDKN1A) [19].

Таким образом, в последние годы накоплены данные о структуре генетических изменений, характерных для пролактин-секретирующих аденом гипофиза. В настоящее время проводится поиск генов, ассоциированных с развитием пролактином, резистентных к терапии агонистами дофамина.

В ходе исследований в значительной части гормонально-активных аденом гипофиза выявлена избыточная экспрессия генов gsp, ccnd1 и PTTG, играющих ключевую роль в клеточной трансформации и пролиферации. Изменения последовательности и структурной организации генов, свойственные именно пролактиномам, включают усиление экспрессии таких генов, как SF3B1, RIS1, POU1F1, POU2F2, DNAJB5, ANGPT1, ELMO1, NOTCH3 и TLE4, ответственных за кодирование различных факторов транскрипции, ростовых факторов и сигнальных пептидов и подавление генов TGFBR3, ST18, DLEU1, IGFBP3, FZD7 [20–24].

Однако исследования генетической структуры репертуара пролактин-секретирующих аденом гипофиза, резистентных к медикаментозной терапии, к настоящему времени ограничены. Так, имеются данные о некоторых мутациях, ассоциированных с резистентностью пролактином к терапии агонистами дофамина. Исследователи под руководством Ch. Li провели секвенирование генома методом whole-genome sequencing (WGS) 21 пациента с пролактиномой, выявив увеличенную частоту мутации гена SF3B1 [20]. Данный факт впоследствии верифицирован путем проведения полимеразной цепной реакции образцов 227 пролактином. Наличие мутации SF3B1R625H индуцирует альтернативный сплайсинг гена рецептора эстрогена (Еstrogen Related Receptor Gamma gene — ESRRG), обладающего чрезмерной аффинностью к транскрипционному фактору Pit-1, что приводит к увеличению экспрессии гена пролактина. В работе Ch. Li продемонстрировано увеличение секреции пролактина при наличии мутации SF3B1R625H, а также усиление митотической активности клеток и снижение апоптоза лактотрофов. Проведенное исследование позволяет предположить роль мутации SF3B1R625H в развитии фармакорезистентности пролактином, а также свидетельствует о ключевом влиянии этого нарушения на клеточный цикл. Кроме того, выполненная работа указывает на возможность определения данной мутации в качестве предиктора ответа на лечение агонистами дофамина и разработки таргетной терапии.

В контексте изучения генетических характеристик резистентных к терапии пролактином отдельного внимания заслуживает исследование под руководством Hua Gao [24]. В ходе работы выявлено 10 вариантов соматических мутаций, отличающихся у резистентных и чувствительных к терапии пациентов. Среди них идентифицирована driver-мутация — гена PRDM2 и выявлено, что уровни мРНК и белка-продукта PRDM2 ниже в случаях аденом, резистентных к терапии. Кроме того, показана ассоциация снижения уровня мРНК PRDM2 с частотой рецидива пролактиномы. Исследователи подчеркивают значимость обнаруженной мутации в формировании резистентности пролактином к терапии, а также предикторную роль в отношении рецидива аденомы. Схематически патогенез возникновения пролактин-секретирующих аденом гипофиза на разных этапах развития органа изображен на рисунке 3.

**Figure:**
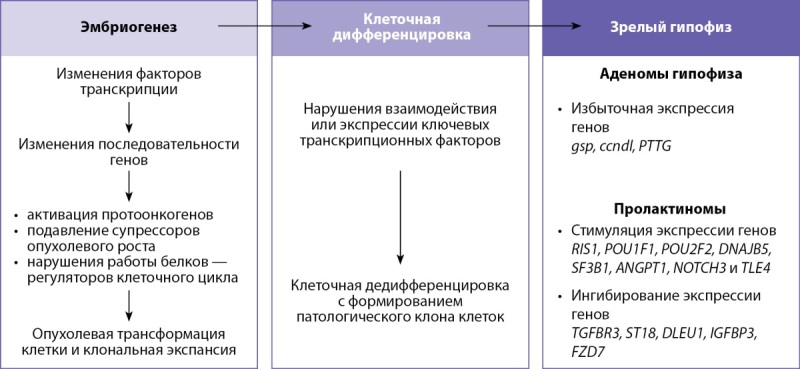
Рисунок 3. Патогенез возникновения аденом на разных этапах развития гипофизаFigure 3. Pathogenesis of adenomas at different stages of pituitary development

Таким образом, изучение молекулярного профиля пролактин-секретирующих аденом гипофиза необходимо для понимания основных механизмов, лежащих в основе резистентности аденом к терапии и опухолевого патогенеза в целом. Наиболее ранняя верификации резистентности к лечению АД с использованием молекулярно-генетических методов позволит на начальном этапе лечения определить оптимальную персонализированную тактику ведения пациента, при необходимости провести конверсию в сторону хирургического метода лечения, предотвратить негативные топографо-анатомические изменения аденомы, снизить риск оперативного лечения.

## ЗАКЛЮЧЕНИЕ

Данные об ультраструктурных особенностях пролактином, как чувствительных, так и резистентных к медикаментозной терапии, ограничены, причины требуют уточнения, а предикторы резистентности отсутствуют. В связи с этим изучение молекулярно-генетических характеристик пациентов с пролактин-секретирующими аденомами гипофиза представляется весьма актуальным для понимания общих патогенетических механизмов резистентности и разработки алгоритма персонализированного ведения пациентов.

## ДОПОЛНИТЕЛЬНАЯ ИНФОРМАЦИЯ

Источники финансирования. Работа выполнена по инициативе авторов без привлечения финансирования.

Конфликт интересов. Авторы декларируют отсутствие явных и потенциальных конфликтов интересов, связанных с содержанием настоящей статьи.

Участие авторов. Шутова А.С. — разработка концепции и дизайна работы, сбор, анализ и интерпретация данных, написание текста; Дзеранова Л.К. — разработка концепции и дизайна работы, проверка критически важного интеллектуального содержания, окончательное утверждение для публикации рукописи; Воротникова С.Ю. — анализ полученных данных, коррекция текста; Кутин М.А. — анализ полученных данных, коррекция текста; Пигарова Е.А. — анализ полученных данных, коррекция текста. Все авторы одобрили финальную версию статьи перед публикацией, выразили согласие нести ответственность за все аспекты работы, подразумевающую надлежащее изучение и решение вопросов, связанных с точностью или добросовестностью любой части работы.
